# Overuse of the psychoactive analgesics’ opioids and gabapentinoid drugs in patients having surgery for nerve entrapment disorders

**DOI:** 10.1038/s41598-023-43253-0

**Published:** 2023-09-27

**Authors:** Lars B. Dahlin, Raquel Perez, Erika Nyman, Malin Zimmerman, Juan Merlo

**Affiliations:** 1 Department of Translational Medicine – Hand Surgery, Skåne University Hospital, Lund University, Jan Waldenströms g 5, 20502 Malmö, Sweden; 2 Department of Hand Surgery, Skåne University Hospital, 20502 Malmö, Sweden; 3 Department of Biomedical and Clinical Sciences, Linköping University, 58183 Linköping, Sweden; 4 Unit for Social Epidemiology, Department of Clinical Sciences (Malmö), Faculty of Medicine, Lund University, 20502 Malmö, Sweden; 5 Department of Hand Surgery, Plastic Surgery and Burns, Linköping University Hospital, 58183 Linköping, Sweden; 6 Center for Primary Health Research, Region Skåne, 20502 Malmö, Sweden; 7 Department of Orthopedics, Helsingborg Hospital, Helsingborg, Sweden

**Keywords:** Risk factors, Neurology, Neurological disorders, Neuropathic pain, Peripheral neuropathies

## Abstract

Knowledge about risks for overuse of psychoactive analgesics in patients having primary surgery for carpal tunnel syndrome (CTS) or ulnar nerve entrapment (UNE), or both, is limited. We investigated if patients with those nerve entrapment disorders have a higher risk of overuse of psychoactive analgesics (i.e., opioids and gabapentinoid drugs) before, after, and both before and after surgery than observed in the general population after accounting for demographical and socioeconomic factors. Using a large record linkage database, we analysed 5,966,444 individuals (25–80 years), residing in Sweden December 31st, 2010–2014, of which 31,380 underwent surgery 2011–2013 for CTS, UNE, or both, applying logistic regression to estimate relative risk (RR) and 95% confidence interval (CI). Overall, overuse of the psychoactive analgesics was low in the general population. Compared to those individuals, unadjusted RR (95% CI) of overuse ranged in patients between 2.77 (2.57–3.00) with CTS after surgery and 6.21 (4.27–9.02) with both UNE and CTS after surgery. These risks were only slightly reduced after adjustment for demographical and socioeconomic factors. Patients undergoing surgery for CTS, UNE, or both, have a high risk of overuse of psychoactive analgesics before, after, and both before and after surgery.

## Introduction

Previous studies have shown an increased risk for opioid overuse after different surgical procedures^1–5^. Following surgery, approximately 10% of patients develop persistent pain, leading to impaired quality of life and eventually to psychiatric conditions, such as anxiety and depression. Unregulated postoperative pain treatment might lead to long-standing, inappropriate use (i.e., overuse) of opioids^6,7^, which in turn may increase the risk of drug dependence and unwanted side effects, including excess morbidity or premature death^8^.

Excessive opioid prescription after surgery has been recognised as an important concern for public health^9^. Yet, the United States and Canada have a seven-fold higher rate of opioid prescriptions filled in the immediate postoperative period compared to Sweden^1^. Even though overuse of opioids in the general population appears to be a much larger problem in the USA than in Sweden^10,11^, there are still concerns about widespread opioid overuse in Sweden as well.

Besides opioid prescription, other psychoactive analgesics, like certain gabapentinoid drugs (i.e., gabapentin and pregabalin), initially indicated as anticonvulsants, are frequently prescribed in postoperative analgesia in hand or orthopaedic surgery as an alternative to opioids. There is, however, evidence recommending against their use, especially in patients with a history of substance use disorders^12^. Therefore, when evaluating long-standing and inappropriate use (i.e., overuse) of pain medication, both opioids and gabapentinoid drugs should be considered.

Concerning surgery for carpal tunnel syndrome (CTS) and ulnar nerve entrapment at the elbow or wrist (UNE), the issue of inappropriate use of analgesics has been scarcely investigated^13,14^. In addition, most previous clinical epidemiological studies are relatively small and based on selected patient samples from specialized clinics, which makes it difficult to compare the overuse of analgesics in treated patients with the general population. In addition, both CTS and UNE surgery are performed on patients suffering from sometimes difficult, long-standing pain, which may severely affect the individuals´ quality of life before surgery^15,16^. This circumstance may increase the risk of inappropriate use of analgesics before surgery. However, long-standing pain, such as neuropathic pain or even Complex Regional Pain Syndrome (CRPS), in CTS and UNE should not be mixed with the more frequently observed paraesthesia or numbness in the innervation area of the affected nerves.

From another perspective, demographic as well as socioeconomic factors may condition both the risk of CTS or UNE and overuse of analgesics. Therefore, a possible association between surgery and use of psychoactive analgesics might be confounded by demographic and socioeconomic factors^10,11,17,18^.

With this background, analysing the whole population of Sweden, our purpose was to investigate the association between surgery for the two most common nerve entrapment disorders CTS and UNE, or the combination of both, and risk of overuse of psychoactive analgesics (i.e., opioids and gabapentin and pregabalin)^12^; before, after, and before and after surgery for these disorders.

## Population and methods

### Databases

The registers of the Total Swedish Population (TPR) and the Longitudinal Integration Database for Health Insurance and Labour Market Studies (LISA), administered by Statistics Sweden (www.scb.se/en/), as well as the National Patient Register (NPR), the Cause of Death Register (CDR) and the Swedish Prescribed Drug Register (SPDR), administered by the National Board of Health and Welfare (www.socialstyrelsen.se/en/), were linked together following an analogous approach as previously described^19^. The record linkage was performed using a unique personal identification number. The procedure was facilitated by the National Board of Health and Welfare and Statistics Sweden after revision and consent by their own data safety committees and initial approval by the Regional Ethical Committee in the South Sweden (#: 2014-856). Data was anonymized by the Swedish authorities before exporting data.

The SPDR contains information about all drug dispensations in the Swedish pharmacies, except from stockpiles in nursing homes and hospital wards, coded according to the Anatomical Therapeutic Chemical (ATC) classification system, while the NPR codes discharge diagnoses from hospital and outpatient clinics according to the International Classification of Diseases and Causes of Death, 10th version (ICD-10). The NPR also records and codes clinical and surgical procedures according to the Swedish version of the NOMESCO Classification of Surgical Procedures (NCSP). The TPR and the LISA database provide demographic and socioeconomic information.

### Population sample

**Figure 1:**
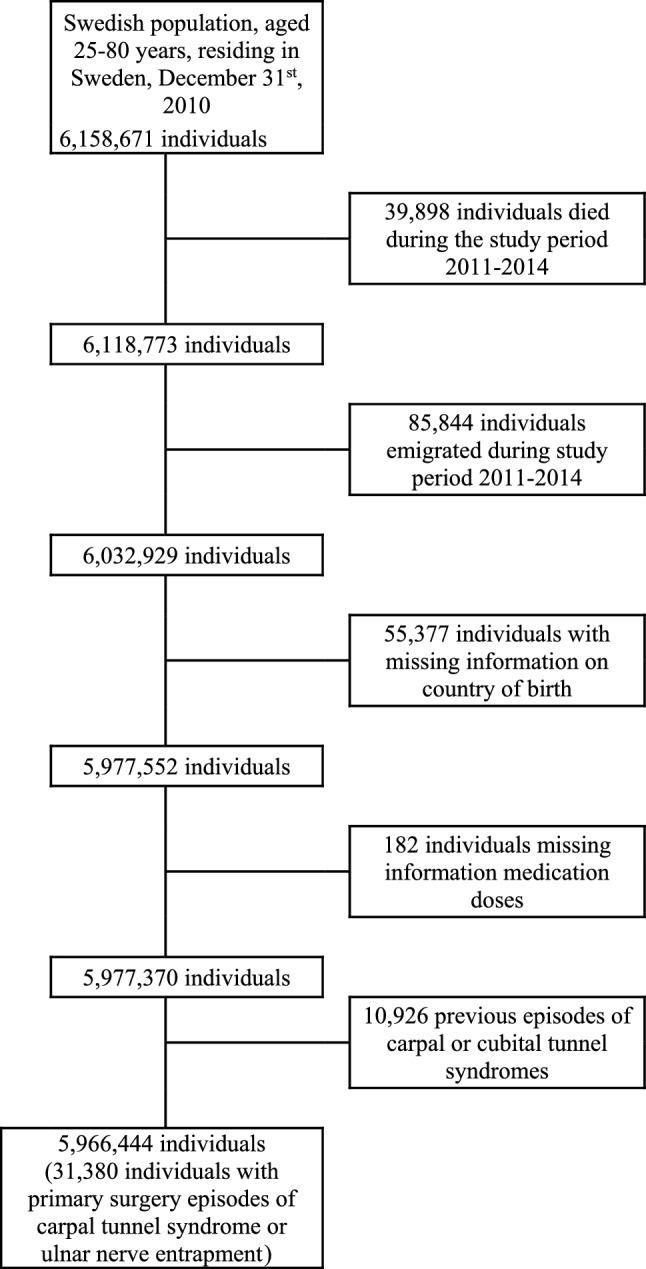
Flowchart showing the population-based and the patient-based samples used in the study. The samples were used for analysing the relation between primary surgery for CTS or UNE, or both, and consumption of psychoactive analgesics (i.e., opioids and the antiepileptic drugs gabapentin and pregabalin).

Firstly, all individuals, aged 25–80 years (appropriate age groups for risk for the present nerve entrapment disorders), residing in Sweden by December 31st, 2010 (n = 6,158,671) were identified. Then, those who died (n = 39,898), emigrated before December 31st, 2014 (n = 85,844), and those without information on country of birth (n = 55,377) as well as those with incomplete information on medication dosage (n = 182) were excluded. Finally, individuals with a previous surgery for the included nerve entrapment disorders during the year before the first surgery episode for such nerve entrapment disorder (n = 10,926) were excluded. The final population dataset consisted of 5,966,444 individuals of whom 31,380 patients presented with a first ever episode of surgery, i.e., primary, for CTS, UNE, or both (see below for variables), during the period 2011–2013. See the flowchart (Fig. 1) for further clarification.

### Assessment of variables

We identified surgery episodes of the nerve entrapment disorders carpal tunnel syndrome (CTS; ICD-10 code G560 and NCSP code ACC51) and ulnar nerve entrapment at the elbow or wrist (UNE; ICD-10 code G562 and NCSP code ACC53). We distinguished between patients suffering from CTS, UNE, and the combination of both, as the clinical experience is that patients with these nerve entrapment disorders present with different clinical profiles that might differentially condition the risk of overuse of psychoactive analgesics.

Most patients with a first surgery episode during the study period 2011–2013 presented specific codes for only one nerve entrapment disorder. If the surgery episode presented codes for both CTS and UNE, we classified the surgery episode as combined CTS and UNE surgery. For every patient with a first surgery episode in the study period 2011–2013, we searched for the existence of episodes within the previous year. If it existed and had a similar code in 2010 the episode was excluded as we tried to identify first-ever surgery episodes during the study period 2011–2013. If the previous episode had a different code, we classified the episode in the study period as combined CTS and UNE surgery. If the patient had a first surgery episode in the study period 2011–2013 and another episode within one year later, we classified the first surgery episode in the study period 2011–2013 as combined CTS and UNE surgery.

Finally, the variable nerve entrapment surgery had the categories, (i) no nerve entrapment surgery, (ii) CTS surgery, (iii) UNE surgery, and (iv) combined CTS and UNE surgery. We used no nerve entrapment surgery (i.e., absence of surgery for nerve entrapment disorders) as the reference in the comparisons (i.e., not considering rare entrapment disorders).

We identified dispensations of opioids according to the ATC codes N02AA01, N02AA05, N02AA55, N02AB01, N02AG02, N02AB03, N02AJ, N02AX, N01AE01, and R05DA04, and of the antiepileptics gabapentin ATC code N03AX12 and pregabalin, ATC code N03AX16. We defined the use of *psychoactive analgesics* as any use of opioids, or the antiepileptics gabapentin or pregabalin or the combination of those drugs. We distinguished between the *use of psychoactive analgesics before surgery*, i.e., during one year prior to the surgery episode of a nerve entrapment disorder as well as the *use of psychoactive analgesics after surgery.* In this case, we considered the use of psychoactive analgesics during one year from the second month from the surgery episode. We did so to avoid the inclusion of any appropriate use of psychoactive analgesics within the closest postoperative period. In individuals without surgery episodes for nerve entrapment, we used 31st December 2010 as the index date. In those periods, we calculated the time covered by the drugs for one year using the cumulative number of defined daily doses (DDDs) of the dispensations divided by 365. We arbitrarily defined the outcome variables *overuse of psychoactive analgesics before, after, and both before and after surgery* as binary variables defined as “1” if the proportion of covered time within the studied year was > 60% and as “0” otherwise. We focused on the psychoactive analgesics’ opioids and gabapentinoid drugs. Therefore, we did not consider other psychoactive drugs not explicitly defined as analgetic drugs.

Age was arbitrarily classified into five wide categories, i.e., 25–34 (reference), 35–44, 45–54, 55–64 and 65–80-year-old. Sex was coded as man (reference) or woman according to the register.

We categorized the individuals according to their country of birth into native (i.e., born in Sweden; reference) or not (i.e., immigrant).

We obtained information on individualised disposable family income for the years 2000, 2005, and 2010 to compute a cumulative measure that considers the size of the household and the consumption weight of the individuals according to Statistics Sweden. For each of the three years, income levels were categorised into 25 groups (1–25) by quantiles using the complete Swedish population. These groups from the respective three years were summed up so that everyone received a value between 3 (always in the lowest income group) and 75 (always in the highest income group). We categorised this cumulative income by tertiles into low, middle, or high (reference) income. Individuals with missing values on income during 2000 or 2005 were assigned the values for the year 2010. No individuals had missing income data for 2010.

Finally, the individuals were categorised into cohabiting as yes (reference) or not according to the LISA database.

### Statistical analyses

We described the demographical and socioeconomic characteristics of the population as well as the existence of overuse of psychoactive analgesics before, after, and before and after surgery by categories of surgery for the nerve entrapment disorders (Table 1).

**Table 1:** Demographical and socioeconomic characteristics of a population with or without nerve entrapment disorders and any overuse of psychoactive analgesics.

	Surgery for CTS, UNE, or combination of both				
	Absence of surgery for nerve entrapment disorders (using 31st Dec 2010 as index date) N = 5,935,064	Presence of any primary surgery for nerve entrapment disorders			
		N = 31,380	CTS N = 28,387	UNE N = 2419	Both N = 574
Overuse of psychoactive analgesics^a^					
Before	50,424 (0.85)	816 (2.60)	659 (2.32)	128 (5.29)	29 (5.05)
After	45,668 (0.77)	848 (2.70)	701 (2.47)	119 (4.49)	28 (4.88)
Before and after	35,149 (0.59)	606 (1.93)	492 (1.73)	94 (3.89)	20 (3.48)
Women	2,963,279 (49.93)	20,649 (65.80)	19,246 (67.80)	1080 (44.65)	323 (56.27)
Age (years)					
25–34	1,100,330 (18.54)	2765 (8.81)	2487 (8.70)	232 (9.59)	46 (8.01)
35–44	1,241,126 (20.91)	5702 (18.17)	5077 (17.88)	494 (20.42)	131 (22.82)
45–54	1,188,590 (20.03)	8217 (26.19)	7359 (25.92)	696 (28.77)	162 (28.22)
55–64	1,155,315 (19.47)	6998 (22.30)	6287 (22.15)	588 (24.31)	123 (21.43)
65–80	1,249,703 (21.06)	7698 (24.53)	7177 (25.28)	408 (16.91)	112 (19.51)
Income					
Low	1,452,850 (24.48)	8354 (26.62)	7564 (26.65)	644 (26.62)	146 (5.44)
Middle	2,061,357 (34.73)	11,727 (37.37)	10,618 (37.40)	883 (36.50)	226 (39.37)
High	2,420,857 (40.79)	11,299 (36.01)	10.205 (35.95)	892 (38.87)	202 (35.19)
Immigrant	961,342 (16.20)	4120 (13.13)	3712 (13.08)	332 (13.72)	76 (13.24)
Cohabiting	3,520,304 (59.31)	19,799 (63.09)	18,164 (63.99)	1278 (52.83)	357 (62.20)

Demographical and socioeconomic characteristics of the population 25–80-year-old and residing in Sweden by December 31st, 2010 and overuse of psychoactive analgesics in relation to absence or presence of surgery for the nerve entrapment disorders (Nerve entrapment disorder = Carpal tunnel syndrome (CTS): according to the International Classification of Diseases (ICD-10 code G56.0) and the NOMESCO Classification of Surgical Procedures (NCSP code ACC51) and ulnar nerve entrapment at the elbow or wrist (UNE) (ICD-10 code G56.2 and NCSP code ACC51), or the combination of both) CTS, UNE or both during 2011–2013. Values are numbers (percentages).

^a^See Assessment of variables for a detailed definition.

Thereafter, we applied logistic regression models to investigate the overuse of psychoactive analgesics. The first model included surgery for only the nerve entrapment disorders. The second model added age, sex, income, country of birth, and cohabiting (Table 2).

**Table 2:** Unadjusted and adjusted relative risks of overuse of psychoactive analgesics for the nerve entrapment disorders.

	Overuse of psychoactive analgesics in relation to surgery for a nerve entrapment disorder					
	Before surgery		After surgery		Before and after surgery	
	Model 1a	Model 2a	Model 1b	Model 2b	Model 1c	Model 2c
	RR (95% CI)	RR (95% CI)	RR (95% CI)	RR (95% CI)	RR (95% CI)	RR (95% CI)
Surgery for nerve entrapment disorder						
No	Reference	Reference	Reference	Reference	Reference	Reference
CTS	3.26 (3.03–3·52)	2.82 (2.62–3.04)	2.77 (2.57–3.00)	2.40 (2.22–2.60)	2.96 (2.71–3.24)	2.54 (2.2–2.78)
UNE	6·67 (5.55–8.02)	5.85 (4.86–7.42)	6.52 (3.46–7.79)	5.76 (4.81–6.89)	6.79 (5.52–8.34)	5.88 (4.78–7.24)
Both	6.61 (4.52–9.67)	5.91 (4.03–8.66)	6.21 (4.27–9.02)	5.57 (3.83–8.12)	6.06 (3.88–9.47)	5.34 (3.41–8.37)
Sex						
Men		Reference		Reference		Reference
Women		1.17 (1.15–1.20)		1.16 (1.14–1.18)		1.17 (1.15–1.19)
Age (years)						
25–34		Reference		Reference		Reference
35–44		2.30 (2.21–2.39)		2.23 (2.15–2.32)		2.50 (2.39–2.62)
45–54		3.23 (3.11–3.36)		3.11 (3.00–3.23)		3.58 (3.43–3.75)
55–64		4.20 (4.05–4.37)		4.05 (3.90–4.19)		4.66 (4.46–4.88)
65–80		3.85 (3.71–4.00)		3.87 (3.73–4.00)		4.14 (3.96–4.32)
Income						
Low		2.55 (2.48–2.61)		2.59 (2.53–2.66)		2.66 (2.58–2.73)
Middle		1.95 (1.90–1.99)		1.95 (1.91–1.99)		2.03 (1.98–2.09
High		Reference		Reference		Reference
Country of birth						
Immigrant		Reference		Reference		Reference
Native		1.28 (1.25–1.31)		1.24 (1.21–1.27)		1.33 (1.29–1.37)
Cohabiting						
Yes		Reference		Reference		Reference
No		1.67 (1.64–1.70)		1.64 (1.61–1.67)		1.68 (1.64–1.73)
AUC	0.51	0.64	0.51	0.67	0.51	0.67

Unadjusted (model 1) and adjusted for demographical and socioeconomic factors (model 2) relative risks of overuse of psychoactive analgesics (i.e., opioids and gabapentinoids) before (a), after (b), and both before and after surgery(c) for the nerve entrapment disorders CTS and UNE, or the combination of both (Nerve entrapment disorder = Carpal tunnel syndrome (CTS): according to the International Classification of Diseases (ICD-10 code G56.0) and the NOMESCO Classification of Surgical Procedures (NCSP code ACC51) and ulnar nerve entrapment at the elbow or wrist (UNE) (ICD-10 code G56.2 and NCSP code ACC51), or the combination of both) (model 1). Values are relative risk (RR) and 95% confidence intervals (CI).

*AUC* area under the receiver operating characteristics curve.

Since the prevalence of the outcome was relatively low, we measured the associations between the explanatory variables and overuse of psychoactive analgesics by relative risk (RR) estimated by odds ratios and 95% confidence intervals (CI) obtained from the logistic regression models.

We estimated the Discriminatory Accuracy (DA) for each model by calculating the area under the Receiver Operating Characteristic curve (AUC). The value of the AUC ranges from 0.5 to 1, with 1 representing perfect discrimination and 0.5 indicating no predictive accuracy. Using the criteria proposed by Hosmer and Lemeshow, we classified DA as absent or very weak (AUC = 0.5–0.6), poor (AUC > 0.6–≤ 0.7), acceptable (AUC > 0.7–≤ 0.8) or excellent (AUC > 0.8–0.90) and outstanding (AUC > 0.90). Stata v14.1 (StataCorp, College Station, TX) was used to conduct the analyses.

### Ethics declarations

Patient consent was waived due to the information consisting of anonymized data obtained from national registers in Sweden as approved by the National Ethical Committee (#: 2014-856). All research was performed in accordance with relevant guidelines and regulations. The research was performed in accordance with the Declaration of Helsinki.

## Results

The demographical and socioeconomic characteristics of the population of 25–80-year-olds, who were residing in Sweden by December 31st, 2010, and overuse of psychoactive analgesics in relation to the absence or presence of surgery for CTS, UNE, or both during 2011–2013, are presented in Table 1. In the population without surgery for these specific nerve entrapments (i.e., absence of surgery), the overuse of psychoactive drugs was very low both before (0.85%) and after (0.77%) December 31st, 2010, and with an even smaller percentage (0.59%) of overuse of psychoactive drugs in both periods. Remarkably, these numbers were higher among patients who had surgery before, after, and before and after surgery for these nerve entrapment disorders (Table 1). More specifically, patients, who had surgery for CTS, presented an overall three times higher overuse of psychoactive drugs before (2.32%), after (2.47%), and both before and after (1.73%) the surgery. However, this increased risk was about six times higher in patients having surgery for UNE or both UNE and CTS before, after, and both before and after surgery (Table 1). The studied patients consisted generally more often of women, except among UNE (more men), and were older than the population without surgery for the specific nerve entrapment disorders. In addition, high-income individuals and immigrants were underrepresented among surgically treated patients. Overall. cohabiting was slightly more frequent among surgically treated patients than in the general population, except for the group of patients having surgery for UNE (Table 1).

Table 2 indicates the results from the regression analyses. In this population-based analysis, model 1a shows that the RR of overuse of psychoactive analgesics *before* surgery is about three times higher in patients with surgery for CTS, and almost seven times higher in those with surgery for UNE alone, or in combination with CTS, than among individuals in the general population. Model 1b shows that the RR of overuse *after* surgery is almost three times higher in patients with surgery for CTS, and more than six times higher in those with surgery for UNE alone, or in combination with CTS, than among individuals in the general population. We found a similar pattern when modeling the overuse of psychoactive analgesics *before and after surgery* in model 1c.

In models 2a, 2b, and 2c, the inclusion of demographic and socioeconomic variables reduced the RR of overuse related to surgery somewhat, as socioeconomic factors may be upstream determinants of both the risk of having surgery for a nerve entrapment disorder and the risk of overusing psychoactive analgesics. Native, women, individuals particularly older than 55 years, individuals having a low income, and individuals living alone presented the highest RR of overuse of psychoactive analgesics. The discriminatory accuracy of models 2a, 2b, and 2c improved as compared with models 1a, 1b, and c, but it remained poor (i.e., AUC between 0.64 and 0.67; Table 2).

## Discussion

Our analysis indicates that in the whole Swedish population, aged 25–80 years and without primary surgery for CTS or UNE, or both, the risk of overuse of psychoactive analgesics (i.e., opioids or gabapentinoid drugs) was low, being less than 1%. However, overall, patients with primary surgery for CTS, UNE, or both CTS and UNE have a much higher risk of overuse before, and after surgery being approximately three times higher in patients with surgery for CTS and between six and seven times higher in patients with surgery for UNE or both CTS and UNE than in the general population. We arbitrarily used a threshold of 60% of the year (i.e., approximately 210 days) for defining the overuse of opioids. This was an arbitrary decision that certainly capture individuals with established long-term use/overuse of opioids, but may underestimate the observed associations. As commented by In-Ae Song et al.^20^, the threshold of long-term opioid therapy in previous publications has ranged from one week to one year, with most of the previous studies using ≥ 90 days of continuous opioid therapy to define long-term opioid use; thus, the present definition of overuse, i.e., a threshold of 60% of the year, was used and applied for the overuse of opioids^21,22^.

The prescription of opioids after surgery for CTS has been highlighted^23^ as a possible risk factor that could trigger the overuse of psychoactive analgesics, which is a general problem and risk in patients with neuropathic pain and CRPS. Our study suggests that suffering from CTS or UNE, and having primary surgery for the conditions, increases the risk of overuse before surgery and this risk withstands after surgery. Several questions are raised concerning the causation of our findings, where the questions are (i) Does the surgery cause pain that requires psychoactive analgesia in the long term? (ii) Does the surgery fail to relieve the pain in a group, or groups, of patients, and then the only treatment is psychoactive analgesia? (iii) Are people with nerve pain who requires psychoactive analgesia more likely to have nerve decompression surgery? (iv) Are patients with pain conditions (e.g., fibromyalgia, sensitization syndromes, etc.) improving after surgery for CTS or UNE, or both, but still have another type of pain that require psychoactive analgesia? We performed a sensitivity analysis to identify if the surgery itself could trigger overuse in some patients. We observed that surgery for CTS, UNE, or both CTS and UNE increased the risk for overuse of psychoactive analgesics after surgery in patients without previous overuse. However, the results of this sensitivity analysis were unreliable because the number of patients meeting the selection criteria was scarce (36 cases of patients receiving surgery distributed as follows: 27 CTS cases, 7 UNE cases, and 2 cases with both procedures). Our study indicates that overuse of psychoactive analgesics is a problem that needs be considered related to the diagnosis of the nerve entrapment disorder(s), the indication for surgery of the disorder(s) as well as for the primary surgery itself for the evaluated nerve entrapment disorders CTS, UNE and the combined disorders CTS and UNE. One should realize that pain is generally not the most prominent symptom among patients with nerve entrapment disorders like CTS or UNE, or a combination of both, although it may be present in some patients. Neuropathic pain should not be mixed with the common “entrapment symptoms”, such as paraesthesia and numbness, which frequently are present in patients with nerve entrapment disorders, particularly preoperatively. To summarize, we were not able to disentangle causal mechanisms behind the described overuse. Our study gives evidence of a high prevalence of overuse of psychoactive analgesics in patients receiving primary surgery for CTS and especially for UNE or both procedures. Our study also shows that overuse of psychoactive analgesics increases with age and is generally increased among those without cohabiting (except in UNE). Furthermore, such overuse is lower among immigrants, and it is higher in women and in individuals with low income. However, the risk of overuse in relation to surgery for CTS, UNE, and the combination of both, remains after adjustment for such demographics and socioeconomic factors.

A decline in opioid prescription has been observed independent of state laws in US^24^. However, inappropriate use of opioids and other psychoactive analgesics, like gabapentinoid drugs, is still a public health problem^25^, irrespective after general surgery^2,3,26^, or after upper limb and hand surgery in particular^4,27,28^. Gabapentinoids are psychoactive, and also sedating, like many other misused prescription medicines, including opioids^8,29^. The prescription of gabapentinoids after surgery for CTS is a problem that has been emphasized with an urge to avoid such a prescription or to stop its use postoperatively^30^. Interestingly, there are signs of an excess prescription of analgesics (e.g., a combination of paracetamol with codeine or paracetamol and tramadol) in relation to the number of tablets of such drugs that the patients in reality consumed during the postoperative period^13^. In the United States, it is recommended that providers use non-opioid analgesics and limit opioid prescriptions after surgery for CTS^14^, which may have implications on the postoperative disability duration and health care costs^6^. On the other hand, patients with previous overuse could be identified and properly treated for such nerve entrapment disorders. Interestingly, a group of patients, referred to a specialized pain management clinic following surgery for UNE, is characterized by prior pain conditions, earlier contact with a pain management clinic, and high degrees of kinesiophobia, depression/anxiety, low quality of life, and low life satisfaction^31^. Such patients have also significantly higher postoperative Disabilities of the Arm, Shoulder, and Hand (DASH) scores, are significantly younger, and have more often bilateral surgery than a reference group^31^. Therefore, the treated surgeon must be particularly careful concerning the indication for surgery among patients with UNE, where also concomitant co-morbidities are described, including affection of the cervical spinal nerve roots. Posttraumatic stress disorder is also described as an independent risk factor for increased use of opioids after surgery for CTS. In contrast, patients with a generalized anxiety disorder have shown a decreased risk for opioid use after such surgery^32^. Other patient characteristics, such as age, sex, ethnicity, having a private insurance, psychological disorders, previous pain diagnosis, history of drug abuse, lower income, low socioeconomic status, mental health disorders, and higher doses, have also been highlighted for postoperative opioid prescription among hand surgeons^18,28,33^, which was confirmed in our study. An association has also been found in patients between those undergoing median and multiple nerve decompression surgery and migraine headache^34^.

In conclusion, pharmacological pain relief is a major problem that needs to be considered in surgery in general and in surgery for CTS, UNE, or both in particular. It appears that over and above demographical and socioeconomic characteristics, patients receiving surgery for CTS, UNE, or the combination of both, have a high risk for overuse of psychoactive analgesics both before, and after surgery, and it could also be possible that surgery triggers overuse in some patients. Therefore, prescription of psychoactive analgesics, such as opioids and gabapentinoids, should be done with caution after a meticulous analysis of the pain problems, and particularly after surgical procedures, like surgery for CTS, UNE, or the combination of both, since there may be a risk for harm if inappropriately prescribed or misused by patients^7^. A better understanding of the epidemiology of surgery-related pain treatment may help to improve quality of care. Early coordination with physicians at pain clinics should be a relevant component in the surgical treatment strategy, especially for patients at high risk of developing severe pain with overuse or long-term use of psychoactive analgesics, e.g., in nerve entrapment disorders.

## Data Availability

Relevant data are included within the paper. The complete and detailed individual data of all subjects cannot be publicly available for ethical and/or legal reasons due to compromising patient privacy. The Regional and National Ethical Committee have imposed these restrictions. The database we analyzed is not publicly available for ethical and data safety reasons. However, the same dataset can be constructed by request to the Swedish National Board of Health and Welfare and Statistics Sweden after approval of the research project by an Ethical Committee (Etikprovningsmyndigheten.se) and by the data safety committees of the Swedish Authorities. The study also needs to be performed in collaboration with Swedish researchers. 1. Public Access to Information Secrecy Act. In: Justice Mo, editor. Stockholm 2009.
